# Watch out where you sleep: nocturnal sleeping behaviour of Bay Island lizards

**DOI:** 10.7717/peerj.1856

**Published:** 2016-04-25

**Authors:** Nitya Prakash Mohanty, Surendran Harikrishnan, Karthikeyan Vasudevan

**Affiliations:** 1 Andaman & Nicobar Environmental Team, Wandoor, Port Blair, Andaman and Nicobar Islands, India; 2 CSIR-Centre for Cellular and Molecular Biology, Laboratory for Conservation of Endangered Species, Hyderabad, Telangana, India

**Keywords:** Predator avoidance, Site fidelity, Tactile cues, Agamid lizards, Tropical islands, Sleeping niches

## Abstract

Sleeping exposes lizards to predation. Therefore, sleeping strategies must be directed towards avoiding predation and might vary among syntopic species. We studied sleeping site characteristics of two syntopic, congeneric lizards—the Bay Island forest lizard, *Coryphophylax subcristatus* and the short-tailed Bay Island lizard, *C. brevicaudus* and evaluated inter-specific differences. We measured structural, microclimatic and potential predator avoidance at the sleeping perches of 386 *C. subcristatus* and 185 *C. brevicaudus*. Contrary to our expectation, we found similar perch use in both species. The lizards appeared to use narrow girth perch plants and accessed perches by moving both vertically and horizontally. Most lizards slept on leaves, with their heads directed towards the potential path of a predator approaching from the plant base. There was no inter-specific competition in the choices of sleeping perches. These choices indicate an anti-predator strategy involving both tactile and visual cues. This study provides insight into a rarely studied behaviour in reptiles and its adaptive significance.

## Introduction

Sleep, a highly prevalent behavioural state across the animal kingdom (Siegel, 2008), has been hypothesised to serve many roles, including energy conservation (Christian, Tracy & Porter, 1984), neural restoration (Siegel, 2003) and predator avoidance (Meddis, 1975; Lima et al., 2005). The long periods of immobility during sleep, along with high intensity of stimulus required for arousal, can make an organism vulnerable to predation. The choice of where an individual sleeps is as important as the phases and duration of sleep (Lima et al., 2005).

Sleeping strategies have received relatively less attention than wakeful behaviours (Siegel, 2003; Lima et al., 2005) and have been largely limited to laboratory experiments with mammals (Stuber et al., 2014). Sleep is characterized by a typical sleep posture, behavioural quiescence, high intensity of stimulus required for arousal and quick reversibility to active state (Tobler, 1985), and is known to occur in reptiles (Flanigan, 1973; Flanigan et al., 1974). Sleeping strategies of ectotherms, such as reptiles, are studied infrequently and mostly by electro-physiological experiments (Ayala-Guerrero & Huitrón-Reséndiz, 1991; Ayala-Guerrero & Mexicano, 2008). Observational studies have been dominated by the polychrotid genus *Anolis* (Goto & Osborne, 1989; Clark & Gillingham, 1990; Shew et al., 2002; Singhal, Johnson & Ladner, 2007; Carbera-Guzmán & Reynoso, 2010). These studies have focused on a few perch characteristics (Goto & Osborne, 1989; Poche, Powell & Henderson, 2005; Reany & Whiting, 2003; Sabo, 2003; Singhal, Johnson & Ladner, 2007; Razafimahatratra, Mori & Hasegawa, 2008; Ikeuchi, Hasegawa & Mori, 2012).

From the limited studies on sleeping behaviour of lizards, it is apparent that sleeping sites can vary across species and local conditions, though a synthesis is lacking. For example, use of sleeping perches which are narrower and less stable than diurnal perches (*Anolis* species, Singhal, Johnson & Ladner, 2007) or higher than diurnal perches (*Acanthocercus atricollis atricollis*, Reany & Whiting, 2003); perches on leaf and branch tips, which may aid in tactile detection of predators (*Lygodactylus tolampyae*, Ikeuchi, Hasegawa & Mori, 2012) and sex-specific choice of thermal microenvironment (Sabo, 2003) have been reported. Differences in sleep sites between two syntopic species (*Anolis* spp., Goto & Osborne, 1989) or lack thereof (*Anolis* spp., Poche, Powell & Henderson, 2005) are also known. A positive relationship between body size and perch height and diameter (*Brookesia decaryi*, Razafimahatratra, Mori & Hasegawa, 2008), an association between diurnal niches and nocturnal sleeping perches (Singhal, Johnson & Ladner, 2007) and variability in head position and orientation with respect to ground (Poche, Powell & Henderson, 2005; Carbera-Guzmán & Reynoso, 2010) have been found.

The influence of predation risk on sleeping strategies (e.g., the choice of sleeping perches) of animals has been inferred through theoretical models (Acerbi & Nunn, 2011) field observations, (Singhal, Johnson & Ladner, 2007; Ramakrishnan & Coss, 2001) and laboratory experiments (Stuber et al., 2014). Experimental studies, on the role of predation in reptilian sleep, have been limited (Mathews et al., 2006; Revell & Hayes, 2009). Apart from its importance in enhancing fitness of an individual, the choice of sleeping perches might lead to competition between syntopic and congeneric species. The preference of species for particular types of sleep sites, such as, structurally unstable perches, high perches or warm perches has been reported. Such preferences could lead to limitation of usable perches and competition. Such competition could impose selection pressures that in turn could result in resource partitioning (Schoener, 1974).

The agamid lizard genus *Coryphophylax* is endemic to the Andaman and Nicobar Islands and has two species, *C. subcristatus* and *C. brevicaudus*. These syntopic diurnal lizards are semi-arboreal and also occupy the forest floor. During the day, they generally perch vertically with respect to the ground, on both narrow and broad tree trunks. Males use diurnal perches to display as part of their territorial and sexual behaviour. *Coryphophylax subcristatus* is found throughout the Andaman and Nicobar Islands, in most forest types including human disturbed areas, while *C. brevicaudus* occurs only in the Andaman archipelago and is limited to dense evergreen and semi-evergreen forests (Das, 1999; Harikrishnan et al., 2012). Among diurnal lizards, *Coryphophylax subcristatus* is the most abundant in the Andaman archipelago, attaining a density of 650 lizards ha^−1^, followed by *C. brevicaudus* at 90 lizards ha^−1^ (Harikrishnan & Vasudevan, 2015). *Coryphophylax subcristatus* is also 1.35 times larger than *C. brevicaudus* (Harikrishnan et al., 2012). Potential nocturnal predators of the lizards include the Andaman cat snake (*Boiga andamanensis*), Andaman pit viper (*Trimeresurus andersonii*), Bay Island wolf snake (*Lycodon hypsirhinoides*), Andaman krait (*Bungarus andamanensis*), and Andaman cobra (*Naja sagittifera*). Potential avian predators are the Andaman barn owl (*Tyto* (*alba*) *deroepstorffi*), Andaman scops-owl (*Otus balli*), Oriental scops-owl (*Otus sunia*), Hume’s hawk owl (*Ninox* (*scutulata*) *obscura*), and Andaman hawk owl (*Ninox affinis*) (Grimmet et al., 2011). Other potential predators include giant centipedes (*Scolopendra gigantea*) and rodents (*Crocidura* spp.).

**Table 1:** Site-wise sampling effort and number of lizards observed. Sampling effort and number of individuals of *Coryphophylax subcristatus* and *C. brevicaudus* observed, across three islands of the Andaman archipelago—South Andaman, Little Andaman and Rutland.

Site	Island	Man-hours	Trails	*C. subcristatus*	*C. brevicaudus*	Total observations
Kanaidera	South Andaman	151.5	24	142	33	175
Wandoor	South Andaman	43.7	9	51	5	56
Mt. Harriet	South Andaman	9.8	1	8	12	20
14 km	Little Andaman	6.0	1	21	0	21
22 km	Little Andaman	11.9	2	41	0	41
Krishnanallah	Little Andaman	20.3	2	24	36	60
South Bay	Little Andaman	7.3	2	50	3	53
Komyo	Rutland	62.8	12	49	26	75
	**Total**	**313.3**	**53**	**386**	**115**	**501**

**Figure 1:**
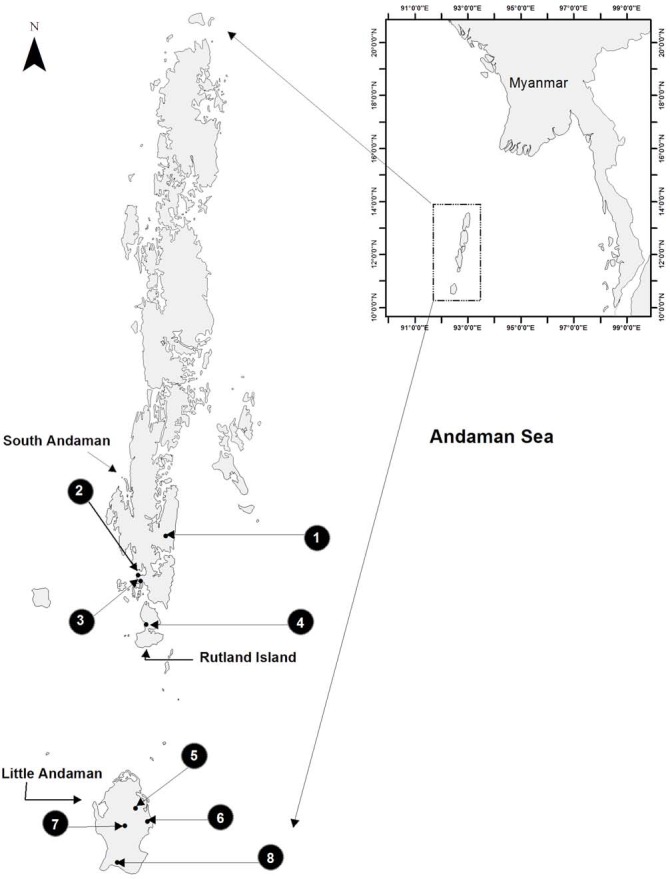
Study area map showing eight sampled sites on three islands of the Andaman archipelago. Sampling carried out in three sites on South Andaman, four sites on Little Andaman and one site on Rutland Island, from September 2014 to January 2015.

We conducted an observational study, to understand sleeping perch characteristics of the genus *Coryphophylax* by considering an exhaustive set of structural, microclimatic and potential predator avoidance measures. We evaluated inter- and intra-specific variations in sleeping perches and preliminarily investigated site fidelity of *Coryphophylax subcristatus*. Additional natural history observations were also recorded.

## Methods

### Study area and effort

**Figure 2:**
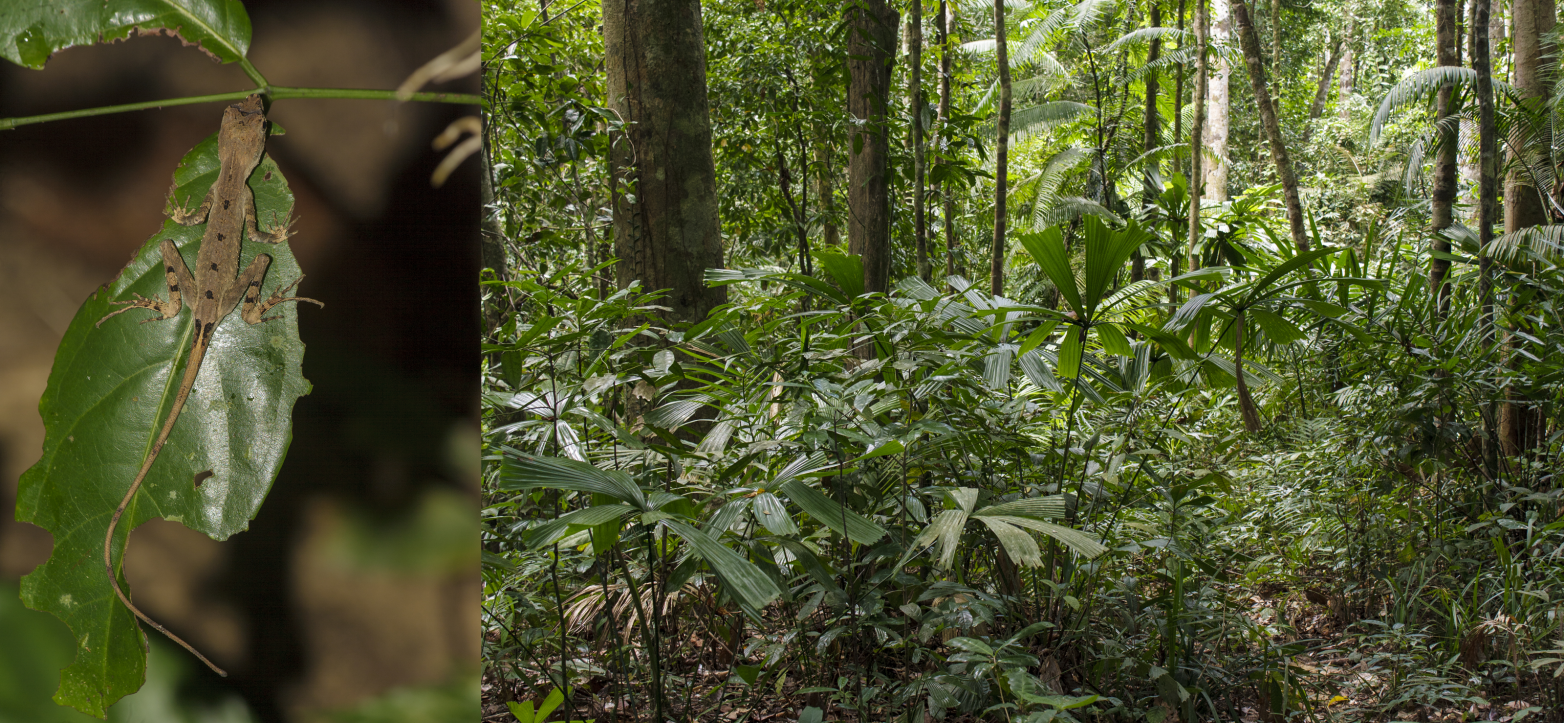
A sleeping *Coryphophylax brevicaudus* and its typical habitat in the evergreen forest. Photo Credit: Harikrishnan S.

We walked 53 trails in eight sites across three islands of the Andaman archipelago: South Andaman, Little Andaman and Rutland, with a total effort of 313.23 man-hours (Fig. 1 and Table 1). We located and measured the sleeping perch characteristics of *Coryphophylax subcristatus* and *C. brevicaudus.* We sampled in the evergreen and semi-evergreen forests representative of the habitats used by the lizards (Fig. 2). All the sites had minimal human disturbance, being part of either reserve forests or National Parks. Department of Forests and Wildlife, Andaman and Nicobar Islands provided permit CWLW/WL/134/350 to conduct fieldwork and handle animals in the Andaman Islands. The study spanned from September 2014 to January 2015. Altitude ranged between ca. 30–650 m. The average night time temperature during sampling was 28.35 ± 0.09 °C with wind speed of 0.03 ± 0.018 km h^−1^ and percentage humidity at 87.75 ± 0.60. The three islands experienced annual rainfall ranging from 3,000 to 3,500 mm (Andrews & Sankaran, 2002). A group of two to four personnel surveyed the forest trails at night with headlamps, searching for sleeping lizards, from the ground to the canopy. Reliable observations could only be made up to a height of 4 m. Forest trails were separated by a minimum distance of 100 m. Searches were not repeated on these trails. We carried out one or more 2-h visual encounter surveys per night, between 1800 h to 0200 h. We did not sample on nights when it rained, as it hampered detection of lizards and accurate measurement. A sleeping lizard was considered as an observational unit.

**Figure 3:**
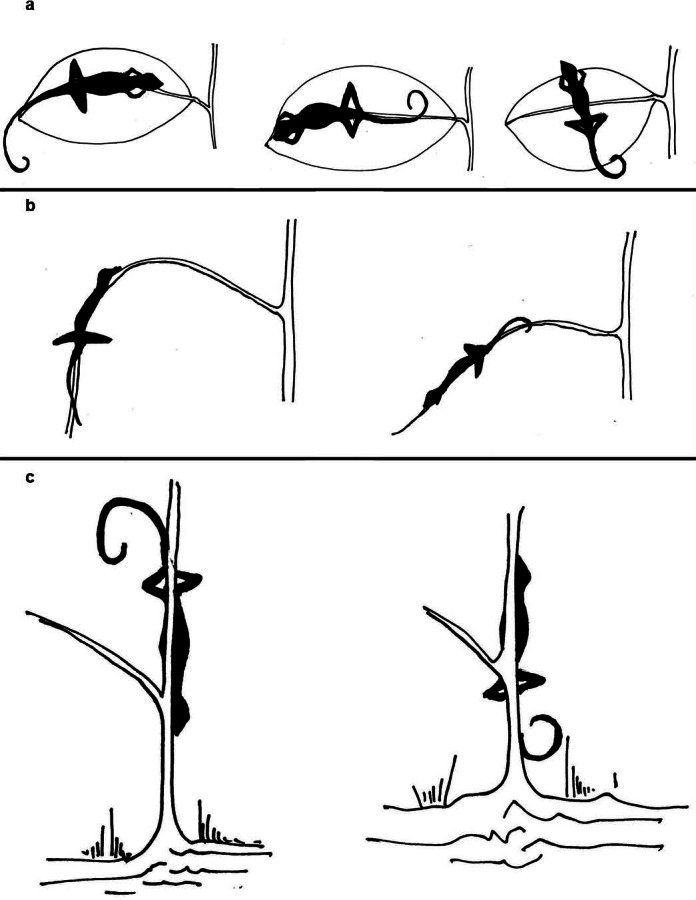
Depiction of head direction of lizards in their sleeping perches. Depiction of head direction of lizards in their sleeping perches, categorized as ‘inward,’ ‘outward’ or ‘perpendicular’ with respect to (A) the petiole, in case of leaf as perch substrate, (B) the trunk, in case of branch as perch substrate and (C) the base of the plant, in case of trunk as perch substrate.

### Perch characteristics and use

On locating a sleeping lizard (Fig. 2), we memorized the perch location, orientation with respect to the ground and head direction of the lizard, to avoid losing data in case the lizard escaped. Then, we proceeded to capture the lizard and marked the head position on the perch. Immediately afterwards, one team member recorded microclimatic measures of the perch, i.e., temperature (°C), percentage humidity and wind speed (km/h), using a pocket weather meter (Kestrel 3000). Simultaneously, one member noted down body size measurements of the lizard, i.e., snout to vent length (SVL), tail length and weight, using a Vernier calliper of 1 mm precision and a Pesola spring balance of 0.2 g precision. All lizards were placed at their original sleeping perch, on completion of the measurements. As there is considerable sexual dimorphism in adult *C. subcristatus* (Harikrishnan et al., 2012), we determined the sex of adult *C. subcristatus* based on morphological features, such as, the presence of dorsal crest in males, size of nuchal crest and colour. Due to low sexual dimorphism in *C. brevicaudus* (Harikrishnan et al., 2012), it was not possible to identify the sexes of this species. We did not attempt sexing by everting hemipenis, as all personnel could not carry out the task with equal efficiency. We measured structural parameters of the perch, i.e., height, leaf length and width (maximum values), maximum girth of trunk (hereafter, girth), branch circumference (at the base of the petiole when the substrate was a leaf) and distance to trunk (non-linear). We noted the orientation of the perching substrate with respect to the ground (horizontal, vertical or angular) and the head direction (inward, outward or perpendicular) of the lizards. We classified the head direction (Fig. 3) with respect to the trunk when the perching substrate was a branch (e.g., inward = head towards the trunk), with respect to the petiole when the substrate was a leaf (e.g., outward = away from the petiole/plant base; perpendicular = across the leaf axis), and with respect to the ground if the substrate was the trunk (e.g., outward = away from the ground). We measured distance to nearest plant in the escape direction of the individual. The escape direction of an individual was assumed to be between 0°to 180°in front of its head (NPM and SH personal observations). The nearest point on the adjacent plant was considered to be on the same plane as or below the lizard. We measured all distances and structural characteristics of the perch using a measuring tape with 1 mm precision.

We also recorded additional variables such as presence of water bodies within a 10 m radius of the perch plant, and plant species (Appendix 1). In this study, selection of sleep sites was not investigated and therefore, availability of sleeping perches was not quantified.

### Site fidelity

To understand fidelity of lizards towards their sleeping site, we marked ten *C. subcristatus* (of all size classes) with roman numerals using blue nail paint. We marked their sleeping sites (on the night of capture) by tying a ribbon (red, 150 mm × 10 mm) to the nearest plant on the left of the perch plant, so as not to change the visual setting. This part of the study was limited to the campus of Andaman and Nicobar Islands’ Environmental Team, South Andaman Island, which has a cover of naturally growing and planted forest. We could not mark *Coryphophylax brevicaudus* as they were unavailable at the site. We searched the area (∼1 ha) for the marked lizards for 13 nights and recorded the distance between each night’s perch and the perch where we had captured them initially.

### Analyses

We performed one-way ANOVAs on normally distributed sleeping perch characteristics (Table 2) to test for differences between the three sampled islands. Kruskal-Wallis rank sum tests were performed to compare variables (Table 2) which did not conform to normal distributions. We pooled the observations from all three islands because the differences were associated with extremely small effect sizes. We assumed that the lizards approached the perch by climbing from the base of the perch plant. Therefore, we computed total distance moved by a lizard as the sum of perch height and distance to trunk. All missing values were left as such and not substituted. As we had fewer data points with measured microclimatic factors (*n* = 164), we analysed that subset of the data separately. We conducted one-way ANOVAs to test for differences between the two species and between the sexes of *C. subcristatus*, in terms of structural and microclimatic measures of the perch. Non-normally distributed variables were compared across these groups by employing Kruskal-Wallis rank sum tests. We constructed multiple regression models to test the relationship between body size and distance, while accounting for the girth of perch plants. We controlled for the effect of body size while evaluating the relationship between sexes of *C. subcristatus* and perch characteristics. The statistical comparisons were carried out at a significance level (*α*) of 0.05.

**Table 2:** Descriptive statistics and inter-specific differences in perch characteristics of *Coryphophylax subcristatus* and *C. brevicaudus*. All measurements have mm as units, except leaf area (mm^2^), temperature (°C), wind speed (m/s) and humidity(%).

	*C. subcristatus*		*C. brevicaudus*					
Characteristics	Mean	CI	Mean	CI	df	*F/H*	*p*	*η*^2^
Girth	59	5.1	41.5	5.0	461	12.77	<0.001	2.60
Branch circumference	12^∗^	1.4	12.6	1.2	494	5.48^∗^	0.019	1.00
Perch height	1100.4	50.4	893	82.7	499	15.65	<0.001	3.04
Distance to trunk	514.7	55.2	358.6	77.9	484	7.92	0.005	1.61
Leaf area	12,478	1,456	7,463	862	348	17.66	<0.001	4.83
Temperature	28.6	0.2	27.73	0.34	162	17.04	<0.001	9.51
Wind speed	0^∗^	0.1	0	0	162	1.15^∗^	0.285	0.70
% Humidity	88.5^∗^	1.57	88.01	1.25	162	0.07^∗^	0.792	0.04
Distance to nearest plant	437.8	26.9	391.4	44.9	466	2.73	0.098	0.50

**Notes.**

Asterisks denote median values and corresponding *H* statistic for non-normally distributed variables.

CI 95% confidence interval df Degrees of freedom *F* Fisher’s F statistic *H* Kruskal-Wallis H statistic *p* Probability value *η*^2^ Effect size in percentage

As the sample size for this study was large (*n* = 501), we relied on effect sizes to infer patterns of biological significance, rather than merely on the basis of *p*-values. For one-way ANOVAs we report effect size−*η*^2^ (Zar, 2010) in percentage.

\begin{eqnarray*}{\eta }^{2}= \frac{\text{Between group SS}}{\text{Total SS}} \times 100 \end{eqnarray*}

where, SS is sum of squared deviance.

We computed leaf area considering a leaf to be an ellipse. Thus, leaf area $=\pi \times (\mathrm{length}\times 0.5)\times (\mathrm{width}\times 0.5)$.

Snout-vent-length was considered as a measure of body size. We calculated effort of a lizard to reach its perch from the base of the plant as, total number of body displacements = total distance moved/SVL.

We report all values in SI units, after converting the raw measurements. All the means reported in the text are provided along with standard error (±SE). We carried out all analyses using the statistical software R (R Core Team, 2013).

## Results

We encountered 386 sleeping individuals of *C. subcristatus* and 115 of *C. brevicaudus*, during our surveys spanning South Andaman, Little Andaman and Rutland islands (Table 1). The encounter rate of sleeping *C. subcristatus* was 3.60 ± 0.41 h^−1^ in South Andaman, 2.85 ± 0.42 h^−1^ in Rutland and 7.71 ± 1.16 h^−1^ in Little Andaman. Corresponding encounter rates of *C. brevicaudus* in the three islands were 0.98 ± 0.22 h^−1^, 0.81 ± 0.27 h^−1^ and 1.73 ± 0.94 h^−1^ respectively.

### Perch characteristics and use

We observed lizards of both species sleeping on different substrates, including leaf, branch, trunk, climber, fallen branch and leaf-branch (body supported by both leaf and branch). We observed both species of lizards on narrow girth (54.8 ± 2.1mm) perch plants with associated structural, microclimatic and potential predator avoidance measures (Table 2). Most lizards of both the species slept horizontally on leaves, with their head directed ‘inwards’ towards the potential path of an approaching predator (Table 3). A majority of *C. subcristatus* (64.24%) slept on leaves, while 87.82 % of *C. brevicaudus* did so.

**Table 3:** Percentage of lizards observed sleeping on different substrates with varied orientation with respect to the ground and head direction. ‘L-B’ refers to both leaf and branch; ‘Other’ includes vines, climbers and adventitious roots. The sample sizes (*n*) refer to observations of ‘orientation’ followed by ‘head direction’.

	*C. subcristatusn* = 384, 368					*C. brevicaudusn* = 112, 111				
Orientation	Leaf	Branch	Trunk	L-B	Other	Leaf	Branch	Trunk	L-B	Other
Horizontal	50.78	9.63	1.04	5.46	1.04	73.21	1.78	0	5.35	0
Angular	11.71	7.55	2.60	1.56	1.04	13.39	0	0	1.78	0
Vertical	2.08	2.08	3.38	0	0	0.89	0.89	1.78	0.89	0

Head direction										
Inward	57.88	8.69	2.44	4.89	0	83.78	1.80	0.90	4.50	0
Outward	4.89	10.05	4.34	1.08	0.54	3.60	0.90	0.90	0	0
Perpendicular	4.07	0	0	1.08	0	0.90	0	0	2.70	0

### Inter-specific and intra-specific variations

**Figure 4:**
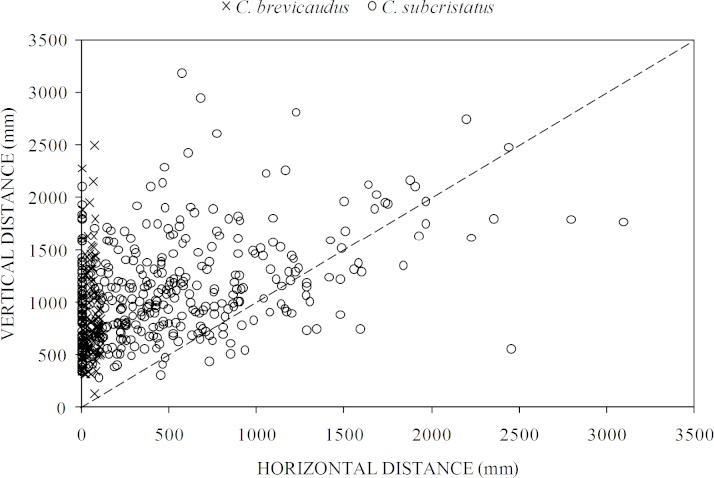
Relative contribution of horizontal distance (perch distance from trunk) and vertical distance (perch height) to the total distance moved. Relationship between horizontal distance (perch distance from trunk) and vertical distance (perch height) in *Coryphophylax subcristatus* (*n* = 372) and *C. brevicaudus* (*n* = 114). The dashed line shows the 1:1 correspondence between the two axes.

We found significant differences in perch characteristics between the two species but effect sizes in all the comparisons were small (Table 2). The two species showed some clear patterns in the way they accessed perches (Fig. 4). While both species moved similar distances vertically, several *C. subcristatus* seemed to access perches away from the trunk (Fig. 4). We found that vertical distance contributed relatively more to the total distance moved than horizontal distance in both species. This pattern was inferred given that most points fell on the upper left margin of the 1:1 correspondence relationship between the two axes in the scatter plot (Fig. 4).

**Figure 5:**
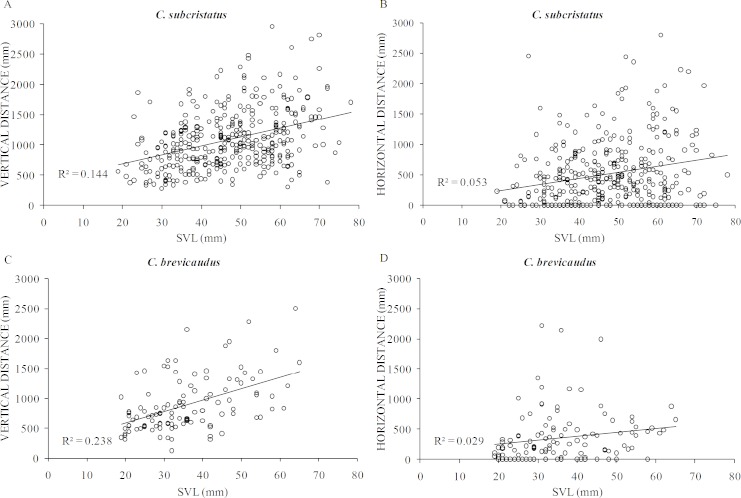
Relationship of body size with vertical distance and horizontal distance. Relationship of body size (snout to vent length—SVL) of individuals belonging to *Coryphophylax subcristatus* (A–B) and *C. brevicaudus* (C–D), with vertical distance and horizontal distance. *R*^2^ denotes the coefficient of determination.

After controlling for girth of perch plants, body size positively influenced vertical distance in *C. subcristatus* (R^2^ = 0.25, *β* = 11.44, SE =1.89, *p* < 0.001; Fig. 5A) and *C. brevicaudus* (*R*^2^ = 0.50, *β* = 11.41, SE =2.81, *p* < 0.001; Fig. 5C). However, body size did not influence horizontal distance moved in *C. subcristatus* (*R*^2^ = 0.35, *β* = 2.69, SE = 1.90, *p* = 0.159; Fig. 5B) and *C. brevicaudus* (*R*^2^ = 0.37, *β* = − 1.96, SE = 2.94, *p* = 0.508; Fig. 5D). We found no difference in effort to reach sleeping perch (i.e., total number of body displacements) between species (F =0.254, df = 476, 1, *p* = 0.615, *η*^2^ = 0.05). Increasing body size was not associated with increasing effort in *C. brevicaudus* (*R*^2^ = 0.01, *β* = − 2.13, SE =1.65, *p* = 0.19) and had a statistically significant but small effect size in case of *C. subcristatus* (*R*^2^ = 0.02, *r* = − 0.14, *β* = − 2.1, SE =0.73, *p* = 0.004).

We found no difference between males and females of *C. subcristatus* with respect to girth of perch plant (*F* = 0.60, df = 166, 1, *p* = 0.438, *η*^2^ = 0.003). After controlling for body size, sex of the lizard did not influence perch height (*R*^2^ = 0.002, *β* = − 0.38, SE =9.34, *p* = 0.966) and perch distance from trunk (*R*^2^ < 0.001, *β* = − 0.73, SE =11.05, *p* = 0.947).

### Site fidelity

We redetected eight out of the ten marked individuals of *C. subcristatus*. The eight individuals were redetected on 3.7 ± 1.02 (1–11) occasions, during 13 nights of observation. We found one adult male (11 nights) and one juvenile (6 nights), sleeping regularly on a specific perch. On average, individuals were found sleeping within a distance of 1.75 ± 0.41 m from their original perch.

### Natural history observations

Most of the lizards of both species were observed sleeping with their eyes open, though this could be an artefact of disturbance by flashlights and motion of personnel. Upon disturbance, the lizards escaped by dropping to the ground and running. When we released the lizards back on their perches, many remained limp and immobile for a few minutes before escaping. We have witnessed the Green bronzeback tree snake (*Dendrelaphis andamanensis*) and Andaman pit viper (*Trimeresurus andersonii*) predating on *Coryphophylax* during the day. We have observed an attempted nocturnal predation, by the Andaman pit viper where the snake was first seen climbing a tall sapling along the main stem. At about 2 m above ground, it started climbing on to a horizontal branch, from which a *Coryphophylax subcristatus* was seen jumping to the ground. We also observed two individuals sleeping on one plant, on four occasions (one and three records of *C. brevicaudus* and *C. subcristatus* respectively). On one such occasion, two *C. subcristatus* (an adult female and a sub-adult) were found sleeping on the same leaf. On another occasion two adult female *C. subcristatus* were observed on top of each other. The lighter individual (SVL = 49 mm and weight = 3 g) was found sleeping on top of the other (SVL = 59 mm, weight = 5 g). We have recorded several instances of juvenile bent-toed gecko, *Cyrtodactylus rubidus*, a nocturnal lizard, sleeping on saplings and one case of the diurnal Andaman skink, *Eutropis andamanensis*, sleeping on a *Pandanus sp*.

## Discussion

### Perch characteristics and use

This study investigates structural, micro-climatic and potential predator avoidance characteristics of sleeping perches in the most abundant genus of lizards in the Andaman archipelago—*Coryphophylax* (Harikrishnan & Vasudevan, 2015). Both species of the genus sleep on narrow girth plants. When compared to their vertical diurnal perches that are mainly on tree trunks, their nocturnal behaviour reveals a tendency to use structurally unstable perches. The narrow girth of perch plants, coupled with extremely thin perch circumference characterize these ‘flimsy’ perches (similar to Shew et al., 2002; Singhal, Johnson & Ladner, 2007; Ikeuchi, Hasegawa & Mori, 2012). The study sites had a tree (>100 mm in GBH) density of ca. 950 ha^−1^ (Mohanty et al., 2016) which would provide many more perches than smaller plants of narrow girth. The almost exclusive use of narrow girth plants (<100 mm in GBH) by the lizards appears to be more than their availability at the study sites. Though, we did not quantify the availability of sleeping perches, the observed pattern suggests selection. This choice of narrow girth plants may discourage heavy predators from climbing the plant.

Most lizards (79%) move vertically and then horizontally away from the base of the plant, attaining a certain distance in each dimension between them and potential predators approaching from the same plant. The benefit of such movement is two-fold: (i) increase in the search time for predators; (ii) increase access to narrow girth perches (Goto & Osborne, 1989). Overall, this sleeping strategy could be explained as a tendency of the lizards to enhance tactile detection and increase search time of predators approaching from the same plant (see Singhal, Johnson & Ladner, 2007). The striking similarity documented in the sleeping strategies of anoline lizards and *Coryphophylax* suggests a possible convergence in such adaptation. Greater observed use of leaves than other substrates as sleeping perches (Table 3) could be due to the larger availability of surface area for traction. Alternatively, it could also be due to pliability of leaves resulting in better tactile detection by the lizards perching on them.

All three microclimatic measures (temperature, humidity and wind speed) showed limited variation during the sampling period. While the effect of wind on sleeping perches inside dense evergreen and semi-evergreen forests may be negligible, changes in temperature and humidity during drier months might influence choice of sleep sites.

A significant role of visual cues probably explains the majority of individuals of both species sleeping with their head directed ‘inwards’–towards the direction of approach of potential predators (Clark & Gillingham, 1990; Carbera-Guzmán & Reynoso, 2010). The importance of visual cues in avoiding predators in the lizards is reinforced from our observations of most lizards found sleeping with their eyes open. Though we do not have quantified data on the nature of eye closure (both or single eye), such behaviour resulting from uni-hemispherical sleep has been reported in reptiles (Mathews et al., 2006; Revell & Hayes, 2009). If such an adaptation occurs, it would allow the lizards to reduce their sleep debt while remaining vigilant (Lima et al., 2005). The distance to nearest plant in the direction of escape was low, as the understory used by the lizards was fairly dense.

### Inter-specific and intra-specific variations

Contrary to our expectation, we did not find pronounced differences between species in structural, micro-climatic and predator avoidance measures. However, the two species accessed horizontally located perches differentially (Fig. 4). The apparent lack of nocturnal resource partitioning is similar to that observed by Poche, Powell & Henderson (2005) but unlike observations of Singhal, Johnson & Ladner (2007) and Goto & Osborne (1989). This suggests that the two species probably partition their niches along other resources, such as, diet and diurnal perches. Specialized use of perches by the two species and both sexes points at an effective sleeping strategy under local conditions.

While there are no biologically meaningful inter-specific differences in perch characteristics, a clear pattern of intra-specific variation emerges. Larger individuals cover greater distances from the base of the plant compared to smaller ones and tend to move more vertically (similar to Clark & Gillingham, 1990; Razafimahatratra, Mori & Hasegawa, 2008; Fig. 5), while there is no difference in horizontal movements. This could be due to the higher energy cost involved in covering greater distances, thereby forcing small individuals to make fewer vertical displacements than large ones. But, considered along with the fact that horizontal movement is not different across body sizes, the relationship of body size with vertical distance could be due to a better climbing ability of large individuals. A lack of difference in perch use between the sexes of *C. subcristatus,* after accounting for body size, discounts any inter-sexual competition leading to contrasting nocturnal perch characteristics.

### Site fidelity

We find evidence of a certain degree of fidelity to sleeping perch in the case of *Coryphophylax subcristatus* (similar to Kennedy, 1959; Clark & Gillingham, 1990; Reany & Whiting, 2003; Singhal, Johnson & Ladner, 2007). It is probable that they use multiple locations within a certain range, to which they return every night. Fidelity to sleeping sites has been explained as choosing of limited high quality perches (Clark & Gillingham, 1990), which maybe ‘safe.’

### Natural history observations

We note a difference in escape strategies during the day and night in both species. Upon disturbance during the day *Coryphophylax spp.* tend to run up tree trunks (SH and NPM personal observations), while at night they usually drop to the ground and escape. This mode of escape is best explained as an avoidance of serpentine predators approaching from the same plant. The option of climbing a great distance up is also curtailed by the narrow-girth perch plants which might be short. Our single observation of an attempted predation on *Coryphophylax* and its escape fits our inference of predator avoidance while sleeping. Our limited observations of females and sub-adults sleeping on the same plant are probably explained by increased combined vigilance (Dehn, 1990) or kinship, but not by social monogamy (Bull, 2000; Harrison, 2013).

## Conclusion

Both species of the genus *Coryphophylax* use structurally and micro-climatically similar sleeping perches. In general, the lizards use perches which are unstable in structure. We infer a role of both tactile and visual cues in detecting and subsequently avoiding nocturnal predators. We did not find evidence of segregation of sleeping perches between the two syntopic and congeneric species and propose investigations on competition for diurnal perch use and food acquisition. While inter-specific and inter-sexual differences are absent, a positive relationship between body size and perch height is apparent. We also found evidence for site fidelity in *Coryphophylax subcristatus*, which may reflect limited number of ‘safe’ perches in the area. Our study provides new insights into a rarely studied behaviour in lizards, the adaptive significance of sleeping behaviour in predator avoidance and resource partitioning in syntopic and congeneric species. Based on reported negative impacts on lizard population by spotted deer (*Axis axis*), an invasive mammalian herbivore in the Andaman Islands (Mohanty et al., 2016), our findings lend support to the importance of understory vegetation structure for long-term survival of these endemic lizards in the Islands.

## Supplemental Information

10.7717/peerj.1856/supp-1 Supplemental Information 1 Key to the data on *Coryphophylax* sleeping behaviour This file contains details of the variables used to collect data on sleeping behaviour of *Coryphophylax* lizards in Andaman Islands.

10.7717/peerj.1856/supp-2 Supplemental Information 2 Raw data on sleeping behaviour of *Coryphophylax* lizards collected from Andaman Islands Raw data on sleeping behaviour of *Coryphophylax* lizards from Andaman Islands used to obtain results in the study.

## Additional Information and Declarations

### Field Study Permissions

The following information was supplied relating to field study approvals (i.e., approving body and any reference numbers): Department of Forests and Wildlife, Andaman and Nicobar Islands provided necessary permits to conduct fieldwork in the Andaman and Nicobar Islands (permit number is CWLW/WL/134/350).

### Data Availability

The following information was supplied regarding data availability: Data can be found in the Supplemental Information.
